# An atypical case of neuronal ceroid lipofuscinosis with co-inheritance of a variably penetrant *POLG1* mutation

**DOI:** 10.1186/1471-2350-13-50

**Published:** 2012-06-24

**Authors:** John F Staropoli, Winnie Xin, Rosemary Barone, Susan L Cotman, Katherine B Sims

**Affiliations:** 1 Department of Pathology, Massachusetts General Hospital, Boston, MA, 02114, USA; 2 Neurogenetics DNA Diagnostic Laboratory, Center for Human Genetic Research, Massachusetts General Hospital, 185 Cambridge Street, Boston, MA, 02114, USA; 3 Molecular Neurogenetics Unit, Center for Human Genetic Research, Massachusetts General Hospital, 185 Cambridge Street, Boston, MA, 02114, USA; 4 Center for Human Genetic Research, 185 Cambridge Street, Boston, MA, 02114, USA

**Keywords:** Neuronal ceroid lipofuscinosis, CLN5, POLG1, mtDNA depletion, Oxidative phosphorylation

## Abstract

**Background:**

The neuronal ceroid lipofuscinoses (NCLs, or Batten disease) comprise the most common Mendelian form of childhood-onset neurodegeneration, but the functions of the known underlying gene products remain poorly understood. The clinical heterogeneity of these disorders may shed light on genetic interactors that modify disease onset and progression.

**Case presentation:**

We describe a proband with congenital hypotonia and an atypical form of infantile-onset, biopsy-proven NCL. Pathologic and molecular work-up of this patient identified *CLN5* mutations as well as a mutation―previously described as incompletely penetrant or a variant of unknown significance―in *POLG1*, a nuclear gene essential for maintenance of mitochondrial DNA (mtDNA) copy number. The congenital presentation of this patient is far earlier than that described for either CLN5 patients or affected carriers of the *POLG1* variant (c.1550 G > T, p.Gly517Val). Assessment of relative mtDNA copy number and mitochondrial membrane potential in the proband and control subjects suggested a pathogenic effect of the *POLG1* change as well as a possible functional interaction with *CLN5* mutations.

**Conclusions:**

These findings suggest that an incompletely penetrant variant in *POLG1* may modify the clinical phenotype in a case of CLN5 and are consistent with emerging evidence of interactions between NCL-related genes and mitochondrial physiology.

## Background

The neuronal ceroid lipofuscinoses (NCLs, also known as Batten disease) comprise a group of at least 10 distinct lysosomal storage diseases with overlapping clinical features including pigmentary retinal degeneration and visual failure in most cases, progressive motor and cognitive decline, epilepsy, movement disorder, and eventual premature death [1]. Eight genes are linked to childhood-onset NCL, but many late-onset clinical forms as well as some pediatric forms remain without identified genetic etiologies. Studies using lower mammal and other eukaryotic disease models suggest that lipid and protein trafficking defects in the endocytic and lysosomal systems underlie NCL disease biology (reviewed in [2-4]).

The pathologic hallmark of most forms of NCL is the lysosomal accumulation of subunit c of the mitochondrial ATP synthase complex [5]. Our previous studies in a genetically accurate model of juvenile NCL (CLN3) demonstrated that this accumulation is preceded by endosomal-lysosomal trafficking defects leading to impaired autophagy as well as by altered mitochondrial morphology, decreased basal ATP levels, and increased sensitivity to oxidative stress [6]. Indeed altered transcriptional regulation of mitochondrial oxidative phosphorylation is an early molecular event in genetically accurate murine cell culture models and may therefore contribute to disease onset or progression [7]. Similar transcriptional alterations have been described in lymphocytes from patients with an autosomal dominant form of NCL caused by mutations in *DNAJC5*, which encodes the synaptic protein cysteine-string protein alpha (CSPα) [8].

Here we describe a patient with an atypical and exceptionally early-onset form of *CLN5*-associated NCL who also carries a variably penetrant dominant mutation (p.Gly517Val) in *POLG1*, a nuclear gene encoding the catalytic subunit of mitochondrial DNA polymerase, which is critical for maintenance of mtDNA copy number and mitochondrial physiology. We present clinical and functional evidence for a possible interaction between an NCL-related gene and a gene involved in mitochondrial homeostasis.

## Case presentation

**Figure 1:**
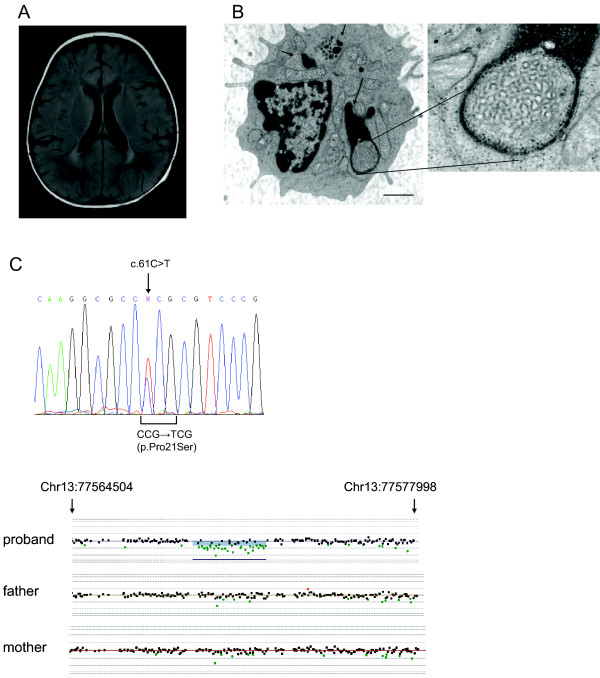
**(A) Cranial FLAIR MRI of the proband at 21 months of age showing mild diffuse cerebral atrophy, particularly in the frontal lobes, and bilateral posterior periventricular white matter changes.** (**B**) Transmission electron micrographs of a representative lymphocyte from the proband showing GROD-like material (arrows) and curvilinear inclusion bodies (inset) characteristic of late-infantile NCL and variant forms. Magnification 26,000X; inset: 105,000X. Scale bar, 1 μm. (**C**) Upper panel: Electropherogram from Sanger sequencing of the proband showing the heterozygous c.61 C > T change in exon 1 of *CLN5*, which predicts a p.Pro21Ser missense change. Lower panel: A portion of the tiling microarray spanning the *CLN5* locus showing a ~2.8 kb one-copy loss (chr13:77569502–77572394) present in the proband but not in either parent. Coordinates reflect human genome build 19.

The proband is the only child of healthy, non-consanguineous parents of northern European descent and was delivered by an uncomplicated Caesarean section for breech presentation at 37 weeks' gestation with a birth weight of 5 lbs, 6 oz. The Apgar score at one and five minutes was 8. The mother had no prior pregnancies. Shortly after birth, the child was noted to have diffuse hypotonia, hypoactive reflexes, and roving eye movements. Height and weight has remained steady at about the 25^th^ percentile, and head circumference continues to be normal. Difficulty tracking visual stimuli was noted at about 4 weeks, and ophthalmologic evaluation has revealed decreased visual acuity, mild bilateral macular pigmentary changes, normal refractive indices, bilateral ptosis, and disconjugate, nystagmoid eye movements. Frequent interruptible staring spells and episodes of lip-smacking and hand-wringing were noted soon after birth. No clear EEG correlate was observed at 5 or 20 months, and no overt myoclonic or generalized tonic-clonic seizures were observed. Evaluation at 4 months of age put her global development at the level of a newborn. MRI imaging at 4 and 21 months showed mild diffuse cerebral atrophy and bilateral posterior periventricular white-matter changes on T2 FLAIR sequences (Figure 1a) without evidence of restricted diffusion by diffusion-weighted imaging. At the time of her most recent examination at 25 months, the patient continued to show the foregoing features and, in addition, failed to initiate motor movements, showed impaired upgaze, and had increased lower extremity tone when placed in upright position but without long-tract signs such as hyperreflexia.

There is no family history of visual failure or seizures, but the proband’s mother does have a distant family history of cognitive delay without clear etiology. Interestingly, the mother herself carries a diagnosis of vitelliform macular dystrophy, a condition associated with lipofuscin accumulation in the retinal pigment epithelium [9]. At the time of the mother’s evaluation, it was unclear whether this retinal process might represent a *forme fruste* of NCL.

## Methods

### Sequencing and multiplex ligation-dependent probe amplification

Total genomic DNA was extracted from peripheral blood specimens using standard manual methods (Qiagen, Valencia, CA). Sanger sequencing of the coding regions and exon-intron boundaries of *CLN3CLN5CLN6MFSD8CLN8*, and *CTSD* was performed on a 3130xl Genetic Analyzer (Applied Biosystems, Foster City, CA). Primer sequences are available on request. Multiplex ligation-dependent probe amplification (MLPA) of the four exons of *CLN5* was performed as described [10].

### Comparative genomic hybridization

Comparative genomic hybridization (CGH) was performed in the Cytogenetics Core of the Dana Farber-Harvard Cancer Center using an oligo-based 60K custom array platform (Agilent Technologies, Palo Alto, California, USA) containing 52,275 probes across the genomic backbone and 553 probes tiling the *CLN5* locus at chr13:77550160–77580653, giving ~50-bp resolution for this region. Probes with unique genomic mappings were used, where possible, and were filtered by predicted melting temperature, unique genomic mappings, repeat content, and for the presence of known SNPs. The DNA was fragmented, labeled, and hybridized according to standard Agilent protocols. The array was scanned on an Agilent Scanner with 2-micron resolution. Intensity values were obtained using Agilent's Feature Extraction software and were subsequently analyzed using Agilent's Genomic Workbench software to identify aberrant regions. Coordinates reflect human genome build 19 [11].

### Quantitative PCR

Relative mtDNA copy numbers from total DNA extracted as above were measured by real-time PCR as described [12] with modifications noted here. DNA was also extracted from fibroblast cultures (described below) grown in 25 mM glucose or 25 mM galactose. Forward and reverse primer sequences for the mtDNA gene target *ND2* were 5'-TGTTGGTTATACCCTTCCCGTACTA-3' and 5'-CCTGCAAAGATGGTAGAGTAGATGA-3'. Forward and reverse primer sequences for the nuclear gene target *GAPDH* were 5'-CATCATCCCTGCCTCTACTG-3' and 5'-TTGGCAGGTTTTTCTAGACG-3'. Real-time PCR was performed using SYBR Green (Roche) according to the manufacturer's instructions and analyzed on a LightCycler 480 instrument (Roche) using the following thermocycling conditions: an initial denaturation step of 95°C for 5 minutes followed by 45 cycles of 95°C for 10 seconds, 56°C for 10 seconds, and 72°C for 10 seconds. Threshold cycle (Ct) values of each target were determined for each individual in triplicate in the same qPCR run. Relative mtDNA copy number was calculated as 2 x (2^ΔCt^), where ΔCt is Ct_GAPDH_ – Ct_ND2_. An unpaired, two-tailed *t* test was used to assess the statistical significance.

### Assay of mitochondrial membrane potential and ETC activity

Fibroblast cultures were derived from skin biopsies obtained under an IRB-approved protocol in accordance with the Helsinki Principles. Subjects used in this study included the proband and an unrelated patient with compound heterozygous *CLN5* mutations (exon 3 c.671 G > A, p.Trp224X and exon 4 c.1103_1106del, p.Lys368SerfsX15), no known mutations in genes related to mitochondrial function, and a typical clinical course, including onset of seizures at age 7. A normal control fibroblast line (GM8330) was obtained from the Coriell Cell Repository (Camden, NJ). Fibroblasts of comparable passage number (4 to 6) were grown under standard conditions in DMEM supplemented with 10% FBS. Relative mitochondrial membrane potential in these cell lines were measured in triplicate (5x10^3^ cells/well in a 96-well plate) with the ratiometric mitochondrial dye JC1 (Cell Technology, Inc., Mountain View, CA) using a fluorescence plate reader (SpectraMax M2, Molecular Devices, Inc., Sunnyvale, CA) as per the manufacturer’s instructions.

Activity of electron transport chain complexes II-IV was measured by established spectrophotometric methods (Center for Inherited Disorders of Energy Metabolism, Case Western Reserve U., Cleveland, OH) in cells grown to confluence under the above conditions.

## Results

Several clinical features in the proband, including possible seizure activity, profound global developmental delay, cortical atrophy, and apparent pigmentary retinopathy prompted an initial diagnostic workup directed towards a form of neuronal ceroid lipofuscinosis. Ultrastructural examination of a buffy coat preparation obtained at 4 months of age showed frequent membrane-bound curvilinear inclusions, material resembling GRODs (granular osmiophilic deposits), and occasional fingerprint-like profiles (Figure 1b and data not shown). Similar findings were present in a concurrent skin biopsy (not shown). GRODs and curvilinear profiles are characteristic of the classic infantile (CLN1) and classic late-infantile (CLN2) forms of NCL, respectively, but may be seen in other forms of NCL [1]. Fingerprint profiles are characteristic of juvenile NCL (CLN3) but may be seen in NCL subtypes, often in combination with other ultrastructural forms. Peripheral blood screening for the enzymes PPT1 and TPP1, deficiencies of which produce CLN1 and CLN2, respectively, was within normal limits (63 pmol/hr [range 21–123] and 51 pmol/hr [range 40–279], respectively; testing performed at the Biochemical Genetics Laboratory of Seattle Children’s Hospital). Sequencing of *CTSD*, which encodes the lysosomal aspartyl protease cathepsin D and underlies a rare congenital form of NCL (CLN10) [13], showed 3 known single nucleotide polymorphisms (SNPs).

Having ruled out the typical earliest forms of NCL, we proceeded to test for variant late-infantile forms of NCL. Molecular analysis of *CLN5* revealed a maternally inherited heterozygous point mutation of a conserved residue in exon 1, c.61 C > T/p.Pro21Ser, and an apparently *de novo* one-copy deletion spanning exon 3 of *CLN5* detected by MLPA and predicted to produce a frameshift and truncated product of 174 amino acids (p.Lys163GlufsX11). The deletion was confirmed by tiling microarray of the *CLN5* locus (Figure 1c). A *de novo* mutation in an NCL-related gene has not been reported to our knowledge. To address the possibility of non-paternity, we completely sequenced the intronic and exonic regions of the *CLN5* gene in the proband and parents and found a SNP pattern compatible with inheritance of the maternal and paternal haplotypes at this locus (data not shown). The c.61 C > T change was absent in over 200 control chromosomes of predominantly European ancestry but was found as an isolated change in a teenage boy with progressive neurologic decline but no further clinical details. The c.61 C > T variant has a carrier frequency of ~0.2% (22/9554 chromosomes) among controls in the Exome Variant Server [14]. This is comparable to the carrier frequency of the more common known pathogenic NCL alleles in the European population. Testing for other NCL-related genes, including *CLN3CLN6, MFSD8*, and *CLN8*, showed no pathogenic changes.

**Figure 2:**
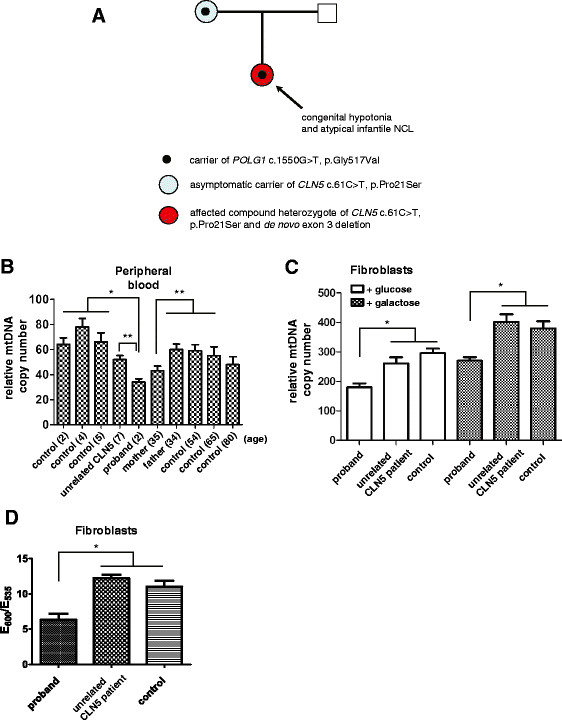
**(A) Pedigree summarizing molecular analysis of*CLN5*and*POLG1*in proband and her parents.** (**B**) Results of real-time PCR analysis of relative mtDNA copy number in peripheral blood from the proband, her parents, an unrelated CLN5 patient with a typical clinical course, and normal controls. Ages are noted in parentheses. Data are shown as the mean ± SEM of triplicate measurements; *, *P* < 0.01, **, *P* < 0.05, two-tailed *t* test. (**C**) Relative mtDNA copy number in cultured skin fibroblasts from the proband, unrelated CLN5 patient, and normal control (GM8330). Fibroblasts were grown under standard conditions (25 mM glucose) or under conditions of metabolic stress (25 mM galactose). Data are shown as the mean ± SEM of triplicate measurements; *, *P* < 0.05, two-tailed *t* test. (**D**) Measurement of relative mitochondrial membrane potential using the ratiometric dye JC-1 in fibroblast lines from the proband, an unrelated CLN5 patient with a typical clinical course, and a normal control subject (GM8330). E_600_ is the emission peak corresponding to normal mitochondrial polarization; E_535_ is the emission peak corresponding to depolarization. Data are shown as the mean ± SEM of triplicate measurements; *, *P* < 0.01, two-tailed *t* test.

A concurrent work-up for the proband’s congenital hypotonia, a feature not typically associated with NCL, included normal testing for Prader-Willi syndrome, spinal muscular atrophy, and an extensive panel of lysosomal storage diseases other than the NCLs. Screens for disorders of amino acid and fatty acid metabolism were also within normal limits. Of note, serum lactate levels were elevated on 2 separate occasions at 2.7 mEq/L and 3.3 mEq/L (range 0.7-1.8 mEq/L), with normal corresponding pyruvate levels. The patient had a normal karyotype, and a targeted 105K microarray (Signature Genomics) showed no pathogenic copy number changes. An extensive screen of mtDNA point mutations was negative, as was a screen for mitochondrial genomic deletions and rearrangements by Southern blotting (testing performed at the Molecular Diagnostics Laboratory of the University of Pittsburgh Medical Center). However, testing of mutations associated with whole mitochondrial genome depletion revealed a heterozygous, maternally inherited change, c.1550 G > T, p.Gly517Val, in *POLG1*, a nuclear gene encoding the catalytic subunit of the polymerase for mtDNA (Figure 2a).

In the absence of a muscle or liver biopsy, we sought to probe the possible functional effect of the *POLG1* mutation by measuring relative mtDNA copy number in peripheral blood and fibroblasts as described previously [12]. Quantitative PCR of a mtDNA gene target (*ND2*) normalized to a nuclear gene target (*GAPDH*) showed an ~45% reduction in the proband in relative mtDNA copy number compared to age-matched controls and an ~30% reduction compared to an unrelated CLN5 patient, described in the methods, with a more typical clinical course (Figure 2b). Furthermore, the carrier mother showed a relative mtDNA copy number lower than that of the father as well as older controls. We also assayed relative mtDNA copy number in subconfluent cultures of fibroblasts from the proband, the unrelated CLN5 patient, and a normal control, GM8330. The proband cell line showed a 30-35% relative reduction in mtDNA both under standard growth conditions (+glucose) and when stressed with a condition that deprives cells of glycolytic capacity and increases reliance on oxidative phosphorylation as an energy source (+galactose) (Figure 2c). Together these results suggest a possible interaction between CLN5 and POLG1 with respect to their effect on mtDNA copy number.

To determine whether these changes in mtDNA copy number might be associated with changes in overall mitochondrial physiology, we assayed mitochondrial membrane potential in the fibroblast lines used above. Basal mitochondrial potential in the proband, as measured by the ratiometric dye JC-1, was reproducibly about half that of the other two cell lines (Figure 2d), consistent with the relative effects on mtDNA copy number seen in Figure 2c. To determine whether this alteration of membrane potential may reflect altered electron transport chain activity, the activities of complexes II, III, and IV were assayed in these same fibroblast lines. Although these activities were within normal limits for all 3 lines (data not shown), we cannot exclude the possible contributions of altered complex I activity, decreased ATP production, and reactive oxygen species production to the lower mitochondrial membrane potential in fibroblasts from the proband.

## Discussion

Each molecular subtype of NCL shows remarkable phenotypic heterogeneity with respect to age of onset, severity of clinical features, and their order of presentation, raising the possibility of genetic modifiers that converge on NCL-associated pathways. Extreme deviations from the usual clinical course may be particularly informative. The case we present here, with onset of symptoms shortly after birth, represents an atypical and unusually early-onset form of CLN5-associated NCL, reported cases of which range in age of onset from 4 to 23 [10]. The presence of additional atypical features in this case, such as congenital hypotonia and nystagmoid eye movements, suggested co-inheritance of modifying factors.

Molecular work-up for these latter features revealed a variably penetrant mutation (c.1550 G > T, p.Gly517Val) in the linker region between the exonuclease and polymerase domains of *POLG1*, a nuclear gene encoding the catalytic subunit of the only known human polymerase that replicates and repairs mtDNA [15]. Most characterized mutations of *POLG1* cause a depletion of total mtDNA and a secondary reduction of the electron transport components necessary for oxidative phosphorylation. Depending on their location in the POLG1 protein, other mutations may additionally cause deletions within the mitochondrial genome or increase the rate of nucleotide misincorporation [16,17]. *POLG1*-related disorders include a spectrum of overlapping and heterogeneous clinical phenotypes including Alpers syndrome (progressive hepatocerebral degeneration, leading to developmental regression, intractable epilepsy, hepatic failure, and death), the ataxia-neuropathy spectrum, spinocerebellar ataxia with epilepsy, mitochondrial neurogastrointestinal encephalopathy, parkinsonism, and progressive external ophthalmoplegia (PEO) [18]. Most of these disorders are inherited in an autosomal recessive manner with the major exception being autosomal dominant forms of PEO.

The p.Gly517Val mutation found in the family described here has been previously reported to manifest as an incompletely penetrant dominant mutation with variable clinical features that include seizures and myopathy ranging in age of onset from one to sixteen years [19]. In one 3-generation family with this mutation, the phenotype was most severe in a subject that also carried, *in trans*, a second variably penetrant substitution, p.Glu1143Gly, in the polymerase domain of POLG1 [20]. Two half siblings heterozygous for p.Gly517Val showed early-onset seizures (one at 13 months of age and the other at 7 years of age), myoclonus, hypotonia, and developmental delay, but their carrier mother was unaffected [21], much as was the case in our family. Together, these findings suggest that other genetic factors elicit or modify the pathogenic effect of the p.Gly517Val substitution and possibly other changes in POLG1.

While we acknowledge that the co-inheritance of the *CLN5* mutations and *POLG1* change in the proband may simply represent an unusual coincidence, the clinical and functional data presented here suggest a genetic interaction between them. Specifically, an incompletely penetrant *POLG1* change (p.Gly517Val) in combination with *CLN5* mutations produced a clinical phenotype in the proband far earlier than that reported for either genetic disorder alone. Furthermore, relative mtDNA copy number in the proband was significantly reduced compared to age-matched controls and an unrelated CLN5 patient with a typical clinical course.

Together, these observations are consistent with accumulating evidence of mitochondrial pathology in the NCLs. Fibroblasts from patients and animal models with certain subtypes of NCL show alterations in mitochondrial morphology, respiratory chain complex activity, and basal ATP synthesis (reviewed in [22]). Gene pathway analysis of altered transcripts in genetically accurate murine cell culture models of CLN3 and CLN6 suggest that dysregulation of oxidative phosphorylation may be a proximal player in these forms of the disease [7]. In addition, human cell lines from CLN5/CLN9 patients show altered transcriptional regulation of multiple subunits of cytochrome *c* oxidase (respiratory complex IV) [23,24]. Finally, fibroblasts from both CLN1 and CLN6 patients show altered mitochondrial distribution, density, and membrane potential, although CLN1 fibroblasts showed only a mild decrease in complex II-III activity and CLN6 fibroblasts showed normal complex I-IV activity [25]. Fibroblasts from the proband described here also showed altered membrane potential without apparent effects on complex activity (specifically complexes II-IV), possibly reflecting compensatory changes of respiratory activity in cultured cells or other contributors to altered membrane potential such as reduced ATP production or dysregulated reactive oxygen species production.

Interestingly, genetic defects in lysosomal storage, such as variants of glucocerebrosidase associated with Gaucher disease [26], and defects in mitochondrial DNA synthesis, including mutations in *POLG1*[27,28], independently contribute to forms of parkinsonism, suggesting that impairments in lysosomal and mitochondrial homeostasis underlie certain shared pathways of neurodegeneration. It will be of interest to probe potential genetic interactions between these pathways in established animal models of NCL [1] and aberrant mtDNA synthesis [29,30].

## Conclusions

We present an atypical and exceptionally early-onset case of CLN5 whose clinical and molecular phenotype may be modified by a variably penetrant mutation, p.Gly517Val, in the linker region of the mtDNA polymerase *POLG1*. Functional in vitro analysis of relative mtDNA copy number and mitochondrial membrane potential supports the pathogenicity of the *POLG1* change and suggest a possible genetic interaction between an NCL-related gene and another involved in mitochondrial homeostasis. Polymorphisms or mutations in genes related to mitochondrial function may represent a novel group of modifiers relevant to the highly heterogeneous NCL disorders.

## Consent

Written informed consent was obtained from the patient’s guardian for publication of this case report and any accompanying images. A copy of the written consent is available for review by the Series Editor of this journal.

## Competing interests

The authors declare that they have no competing interests.

## Authors’ contributions

KBS and JFS were responsible for the clinical and molecular diagnostic workup of the family. JFS and SLC conceived of the functional studies and interpreted the results derived from them. WX and RB contributed to the molecular diagnostic workup. JFS drafted the manuscript, and SLC and KBS revised it. All authors read and approved the final manuscript.

## Pre-publication history

The pre-publication history for this paper can be accessed here:

http://www.biomedcentral.com/1471-2350/13/50/prepub
